# In Vitro Digestion Influences Bioactive Compound Profiles, Antioxidant Activity, and Free Amino Acid Release in Plant-Based Milk Alternatives

**DOI:** 10.3390/foods15162907

**Published:** 2026-08-19

**Authors:** Nonthiwat Taesuk, Theeraphan Chumroenphat, Nidthaya Seephua, Manmanut Srikaew, Sirithon Siriamornpun, Daniela Barile, Apichaya Bunyatratchata

**Affiliations:** 1 Department of Biology, Faculty of Science, Mahasarakham University, Kantarawichai 44150, Maha Sarakham, Thailand; nonthiwat.t@msu.ac.th; 2 Sustainable Approaches for Materials, Agriculture and Health Technology (SAMAHT) Research Unit, Mahasarakham University, Kantarawichai 44150, Maha Sarakham, Thailand; 3 Cosmetic Science and Spa Program, Faculty of Thai Traditional and Alternative Medicine, Ubon Ratchathani Rajabhat University, Mueang Ubonratchathani 34000, Ubonratchathani, Thailand; theeraphan.c@ubru.ac.th; 4 Department of Food Technology and Nutrition, Faculty of Technology, Mahasarakham University, Kantarawichai 44150, Maha Sarakham, Thailand; nidthaya.s@msu.ac.th (N.S.); manmanut@doeb.go.th (M.S.); sirithon.s@msu.ac.th (S.S.); 5 Research Unit of Thai Food Innovation (TFI), Mahasarakham University, Kantarawichai 44150, Maha Sarakham, Thailand; 6 Department of Energy Business, Ministry of Energy, Si Racha 20230, Chonburi, Thailand; 7 Department of Food Science and Technology, University of California Davis, Davis, CA 95616, USA; dbarile@ucdavis.edu

**Keywords:** phenolic acids, flavonoids, simulated gastrointestinal digestion, plant-based beverages, enzymatic digestion

## Abstract

This study evaluated the bioactive compounds, antioxidant activities, phenolic and flavonoid profiles, and amino acid composition of six plant-based milk alternatives (coconut, almond, cashew nut, soybean, peanut, and mung bean) and assessed their changes following in vitro digestion. Mung bean milk exhibited the highest total phenolic and flavonoid contents and antioxidant activity among all samples. Generally, enzymatic digestion significantly increased total phenolic content and antioxidant activity across all plant-based milks, with DPPH activity increasing approximately 2–4-fold, while total flavonoid responses were mostly unchanged. Individual phenolic compounds, including gallic, syringic, and gentisic acids, were newly detected after digestion in several plant-based milks. Protocatechuic acid, which was present only in trace amounts in a few controls, increased more than 200-fold after enzymatic digestion, indicating enhanced release of matrix-bound compounds. Free amino acid levels also increased following digestion, particularly in almond, cashew nut, and mung bean milks, whereas coconut, soybean, and peanut milks showed minor changes. These findings highlight varied nutritional and functional responses to digestion among plant-based milks, providing a basis for the development of functional beverages.

## 1. Introduction

In recent years, the global food system has experienced a profound shift, marked by increasing consumer movement away from animal-based products toward plant-derived alternatives. This trend has been largely influenced by increased health awareness, ethical concerns, and the demand for more sustainable food choices that minimize environmental impacts [1]. Among the wide variety of plant-based foods, milk alternatives have grown rapidly, particularly in the field of functional beverages [2,3]. Several of these products are now commercially available, and research investigating their properties has increased substantially in recent years [4,5].

Plant-based milk alternatives are liquids produced by reducing plant materials to fine particles, extracting them with water, and homogenizing the mixture to mimic the appearance of cow’s milk [6]. Although these products are generally low in protein content compared to cow’s milk except for soy milk [7], these beverages are favored by consumers for various reasons, including health-related needs (e.g., lactose intolerance, milk allergies) and dietary preferences (vegetarian or vegan diets), and cultural, religious, and environmental motivations [8]. While the nutritional value of plant-based milk alternatives may be lower in certain aspects compared to cow’s milk, they are rich in bioactive compounds, such as phenolics and flavonoids, which exhibit antioxidant, anti-inflammatory, and protective effects against chronic diseases, thus enhancing their functional food value and potential health benefits for consumers [6,9,10,11,12].

In recent years, several popular plant-based milk alternatives, including soy, almond, coconut, peanut, rice, oat, sesame, and flaxseed milk, have been investigated [5,6,12]. However, comprehensive studies detailing the effects of digestion on bioactive compounds at the level of individual molecules are still limited. Simulated gastrointestinal systems (in vitro digestion models) are commonly used to examine the release, transformation, and absorption of these compounds, providing important insights into their potential health benefits [13]. Most existing research on the effect of digestion in plant-based milk alternatives has focused on protein digestibility, lipid digestion, and bioaccessibility of vitamins and minerals [14,15,16,17]. Although some studies have examined changes in bioactive compounds and antioxidant activity during digestion, these reports typically address overall levels rather than profiling individual compounds in plant-based milks [16,18]. Digestive conditions, pH, digestive enzymes, and matrixes can influence the release, stability and bioactivity of bioactive compounds. In general, there has been little research on how digestion influences bioactive compounds in plant-based milks, highlighting the need for further investigation. In addition, free amino acids in these beverages may also be modified by digestive enzymes, pH fluctuations, and other gastrointestinal conditions. These modifications can either diminish or, in some cases, enhance their biological activity, and therefore require further investigation.

Based on differences in plant matrix composition, this study hypothesized that enzymatic digestion may influence the release of certain bioactive compounds, antioxidant activity, and free amino acid composition, with responses varying among different plant-based milk alternatives. Therefore, this study aims to investigate the bioactive compounds, antioxidant activities, and amino acid profiles of six plant-based milk alternatives, including coconut, almond, cashew nut, soybean, peanut and mung bean, and to evaluate their changes during in vitro digestion. Since some of these plant-based milks, especially mung bean, remain underexplored, their general profiles were also examined. Accordingly, the research was conducted in two parts. The first part assessed overall bioactive characteristics, including total phenolic content (TPC), total flavonoid content (TFC), antioxidant activities, and total amino acid profiles. The second part examined the effects of digestion on these parameters and further analyzed individual phenolic and flavonoid compounds, as well as free amino acids, after digestion. These investigations not only provide deeper understanding of the underlying mechanisms but also support the development of next-generation functional beverages that promote consumer well-being and align with sustainable dietary practices.

## 2. Materials and Methods

### 2.1. Assessment of Bioactive Compounds, Antioxidant Activities, and Amino Acid Profiles in Plant-Based Milk Alternatives

#### 2.1.1. Preparation of Plant-Based Milks from Raw Materials

Plant-based milk alternatives were prepared using the previous published method [19]. Briefly, six types of raw materials, including coconut, almond, cashew nut, soybean, peanut, and mung bean, were sourced from the same production batch to minimize variability. Prior to processing, all seed materials were washed thoroughly and soaked in deionized water at a ratio of 1:9 (*w*/*v*) for 24 h at ambient temperature. The hydrated samples were ground, and the resulting slurry was filtered through cheesecloth to obtain the milk extract. The filtrates were pasteurized at 80 °C for 5 min, rapidly cooled, and stored at −20 °C until further analysis.

#### 2.1.2. Color Measurement of Six Plant-Based Milk Alternatives

The color of the six plant-based milk samples was analyzed using a Chroma Meter CR-400 (Konica Minolta Inc., Osaka, Japan) in accordance with the manufacturer’s instructions. Results were expressed in the CIE L* a* b* color space. All measurements were conducted in triplicate. Color was evaluated only in the original plant-based milk samples, as this parameter is primarily related to product appearance and consumer perception before digestion; therefore, post-digestion color analysis was not included in this study.

#### 2.1.3. Characterization of Bioactive Compounds in Plant-Based Milk Alternatives

Plant-based milk samples (30 mL) were defatted by centrifugation at 7000 rpm for 10 min at 4 °C (Andreas Hettich GmbH & Co. KG, Tuttlingen, Germany). The experiment was conducted in triplicate, and the skim fractions collected from the three replicates were combined to obtain a stock solution for further determination of total phenolic content (TPC), total flavonoid content (TFC), and antioxidant activity.

Total phenolic content (TPC) was determined using the Folin–Ciocalteu assay following the method described previously [19]. Briefly, 0.5 mL of the sample was combined with 2.5 mL of 10% Folin–Ciocalteu reagent and allowed to react for 4 min. Subsequently, 2 mL of 7.5% sodium carbonate solution was added, and the mixture was incubated in the dark at ambient temperature for 2 h. Absorbance was measured at 725 nm using a spectrophotometer. Results were expressed as micrograms of gallic acid equivalents per milliliter of sample (µg GAE/mL). The assay was performed in triplicate using separate aliquots from the stock solution.

Total flavonoid content (TFC) was analyzed according to a previously reported method [19]. In brief, 500 μL of the sample was mixed with 2.25 mL of deionized water and 150 μL of 5% NaNO_2_ solution. The mixture was kept in the dark for 6 min, then 300 μL of 10% AlCl_3_ was added and incubated for another 6 min, followed by the addition of 1 mL of 1 M NaOH and 550 μL of deionized water. Absorbance was recorded at 510 nm using a spectrophotometer. TFC values were expressed as micrograms of quercetin equivalents per milliliter of sample (µg QE/mL). All analyses were carried out in triplicate using separate aliquots from the stock solution.

Antioxidant activity was determined using the 2,2-diphenyl-1-picrylhydrazyl (DPPH) radical scavenging assay following a previously described method [19]. Briefly, 0.5 mL of the sample was combined with 4.5 mL of 0.06 mM DPPH solution, mixed thoroughly, and incubated in the dark at ambient temperature for 30 min. The absorbance was then recorded at 517 nm, and the antioxidant activity was expressed as micrograms of vitamin C equivalents per milliliter of sample (µg VCE/mL). The assay was performed in triplicate using separate aliquots from the stock solution.

The ferric reducing antioxidant power (FRAP) assay was performed according to a previously reported method [19]. In brief, 0.1 mL of the sample was mixed with 4.5 mL of FRAP reagent, incubated in the dark at ambient temperature for 10 min, and the absorbance was measured at 593 nm. Antioxidant capacity was expressed as micrograms of ferrous sulfate per milliliter of sample (µg FeSO_4_/mL). All measurements were carried out in triplicate using separate aliquots from the stock solution.

#### 2.1.4. Quantification of Total Amino Acids by LC–MS/MS

Following a previously described method [19], 0.7 g of plant-based milk samples were mixed with 5 mL of 6 N HCl. Hydrolysis was carried out in a hot-air oven (WTB Binder ED115/E2, WTB, Tuttlingen, Germany) at 110 °C for 23 h. The acid solution was then removed using rotary evaporation (Buchi R-210, Buchi, Flawil, Switzerland). The samples were subsequently redissolved in 10 mL of distilled water, filtered through a 0.22 μm nylon membrane, and analyzed by LC–MS/MS. The analysis was performed in three replicates, and the results were expressed as μg/g sample.

Data visualizations were performed using R software version 4.5.1. A heatmap of total amino acids was generated using the heatmaply package [20] to illustrate overall amino acid profiles and compositional differences among samples.

### 2.2. Effect of In Vitro Digestion on Bioactive Compounds, Phenolic and Flavonoid Profiles, Antioxidant Activities, and Free Amino Acids in Plant-Based Milk Alternatives

#### 2.2.1. In Vitro Digestion Method

In vitro digestion was performed based on the standardized static INFOGEST protocol [21,22], with minor modifications. The prepared plant-based milk samples were stored under the same conditions at −20 °C for the same storage period until in vitro enzymatic digestion. All samples were thawed only when required for the digestion experiment to minimize repeated freeze–thaw cycles. As the samples were in liquid form, the digestion process was initiated directly from the gastric phase [22]. Briefly, 2 mL of each plant-based milk was mixed with simulated gastric fluid (SGF) and pepsin at a ratio of 1:1 to obtain a final gastric volume of 4 mL (final pepsin activity: 2000 U/mL) [21]. The samples were incubated at pH 3 and 37 °C with constant shaking for 2 h. Following the gastric phase, simulated intestinal fluid (SIF), pancreatin (final trypsin activity: 100 U/mL) and bile salts (10 mM) were added at a ratio of 1:1 to achieve a final intestinal volume of 8 mL [21]. The samples were incubated at pH 7 and 37 °C for an additional 2 h. The control samples were performed following the same procedure as the experimental samples, but without the addition of digestive enzyme.

To terminate enzymatic activity at the end of the intestinal phase, four volumes of cold ethanol (−20 °C) were added to each sample. The mixtures were kept at −20 °C for 24 h then centrifuged at 16,000× *g* for 15 min at 4 °C. The supernatants of digested samples were collected and stored at −20 °C until further analysis. These samples were subsequently analyzed for TPC, TFC, antioxidant activities measured by DPPH and FRAP assays, individual phenolic acid and flavonoid profiles, and free amino acid composition. For each sample, three separate aliquots were prepared and independently subjected to the complete in vitro digestion procedure.

#### 2.2.2. Determination of TPC, TFC and Antioxidant Activities Measured by DPPH and FRAP

The TPC was determined following a published protocol [23]. The absorbance was measured at 725 nm, and TPC was expressed as mg gallic acid equivalents per mL of sample (mg GAE/mL). The TFC was assessed according to a previously described method [23]. The absorbance was read at 510 nm, and results were reported as mg of quercetin equivalents per mL of sample (mg QE/mL).

The antioxidant capacity of the extracts was evaluated using the DPPH assay according to a published method [24]. The absorbance was measured at 517 nm and the scavenging capacity was expressed as µg vitamin C per mL of sample (µg vitamin C/mL). The FRAP assay was performed following the described protocol [19]. The absorbance was measured at 593 nm. Antioxidant capacity was expressed as mg ferrous sulfate equivalents per milliliter of sample (mg FeSO_4_/mL). All measurements were carried out in triplicate from independent digestion experiments.

#### 2.2.3. Determination of Phenolic Acids and Flavonoids

Individual phenolic acid and flavonoid profiles were determined in both control samples without digestive enzymes and experimental samples subjected to enzymatic digestion. The samples were analyzed using the HPLC system (Shimadzu, Kyoto, Sciences Inc., Tokyo, Japan) and a guard column (Inertsil ODS-3, 5 µm, 4.0 × 10 mm). The analysis was performed according to a previously described method [25]. The detection wavelengths were 280 nm, 320 nm and 370 nm for hydroxybenzoic acid, hydroxycinnamic acids and flavonoids, respectively. The extracted samples were identified using external standards of phenolic acids and flavonoids. Each sample was analyzed in three replicates from independent digestion experiments, and the results were expressed as µg/mL sample.

#### 2.2.4. Determination of Free Amino Acids by LC-MS/MS

The digested samples were filtered through a 0.22 μm nylon membrane. Free amino acids were quantified using a Shimadzu LCMS-8030 triple quadrupole mass spectrometer equipped with an electrospray ionization (ESI) source and coupled with a Shimadzu HPLC system (Shimadzu, Kyoto, Japan). Chromatographic separation was performed on an InertSustain^®^ C18 column (2.1 × 150 mm, 3 μm) with a guard column (InertSustain C18, 3 μm, 2.1 × 10 mm). The mobile phase, gradient conditions, flow rate, and MS parameters were applied according to a previously published procedure [19]. Data acquisition was performed in multiple reaction monitoring (MRM) mode. Quantification was based on calibration curves constructed from standard amino acids. All measurements were carried out in triplicate from independent digestion experiments and results were expressed as μg of amino acid per melilite of sample (μg/mL).

Two heatmaps were constructed to examine patterns in free amino acid composition across treatments. First, a heatmap of free amino acids was generated to visualize absolute concentration differences between control (no enzyme) and experimental (enzymatic digestion) samples, allowing direct comparison of quantitative changes. Second, to facilitate comparison of relative abundance patterns and identify dominant free amino acids within each sample, the free amino acid data were standardized using z-score normalization prior to visualization. Z-score transformation centers the data by subtracting the mean from each variable and scaling by its standard deviation. This approach is particularly appropriate for datasets where compounds can differ substantially in concentration ranges [26,27], enabling the identification of features that are relatively enriched or depleted across samples.

### 2.3. Statistical Analysis

All experiments were conducted in triplicate. Data are presented as mean ± standard deviation (SD). Data quality was assessed prior to analysis, and values considered to be anomalous due to apparent analytical or measurement error were excluded. Statistical analysis was performed using IBM SPSS Statistical Software version 17.0. One-way ANOVA was applied, and Duncan’s multiple range test was used to determine significant differences at a 95% confidence level (*p* < 0.05).

## 3. Result and Discussion

### 3.1. Characterization of Bioactive Compounds, Antioxidant Activities, and Amino Acid Profiles in Plant-Based Milk Alternatives

#### 3.1.1. Appearance and Color Characteristics of Plant-Based Milks

**Figure 1:**
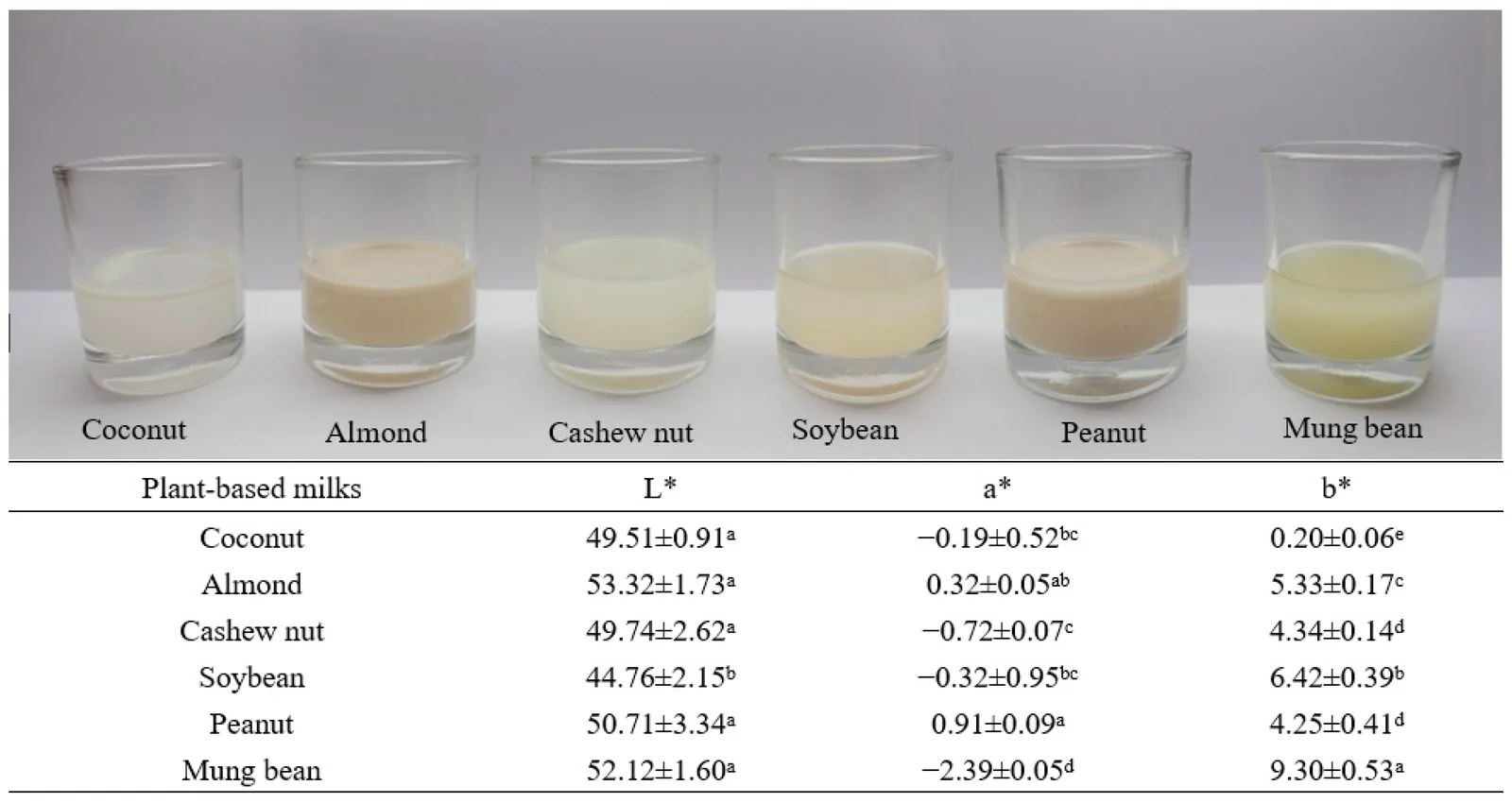
Appearance and color characteristics (CIE L*a*b*) of six plant-based milk alternatives. Different superscript letters within the same column indicate significant differences among plant-based milk alternatives for each color parameter (*p* < 0.05).

The appearance and color characteristics (L*, a*, b*) of six plant-based milk alternatives, including coconut, almond, cashew nut, soybean, peanut, and mung bean, are presented in Figure 1. Color parameters were defined using the CIELAB system as lightness (L*), the green–red axis (a*), and the blue–yellow axis (b*) [28]. Color attributes varied among samples, with L* values ranged from 44.76 to 53.32, a* values from −2.39 to 0.91, and b* values from 0.20 to 9.30. Mung bean milk exhibited the most greenish hue (a* = −2.39) and the most intense yellow tone (b* = 9.30), reflecting the presence of natural pigments of mung bean [29]. Almond milk and peanut milk displayed similar appearances, showing no significant differences in L* and a* values, although slight variation was observed in b*. Both samples showed positive a* values, indicating a greater degree of redness compared to the others. In contrast, coconut, cashew nut, soybean, and mung bean milk alternatives exhibited negative a* values. Although some differences were not statistically significant, these samples generally appeared greener than almond and peanut milk alternatives (Figure 1). Color was assessed only in the original samples because it primarily reflects product appearance and consumer perception before digestion; therefore, post-digestion color analysis was not performed.

#### 3.1.2. Total Phenolic Content, Total Flavonoid Content, and Antioxidant Activities

**Figure 2:**
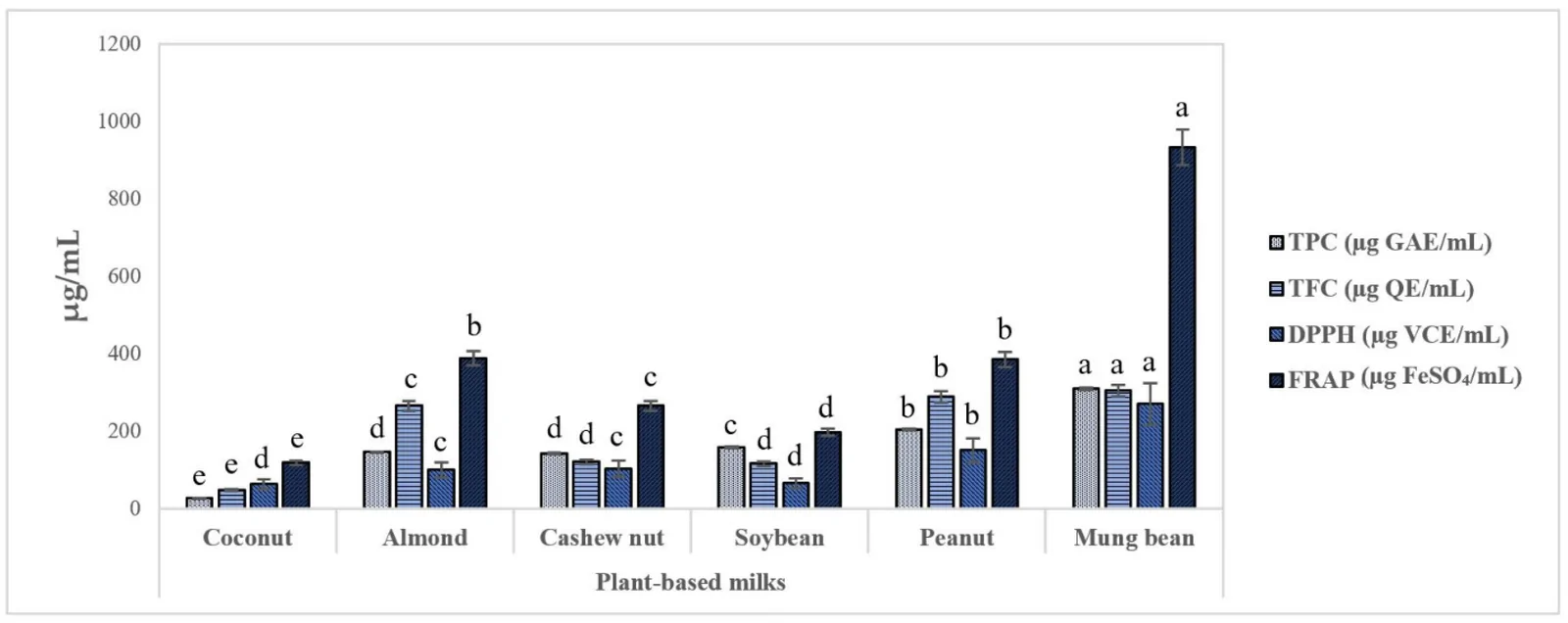
Total phenolic content (TPC), total flavonoid content (TFC), and antioxidant activities measured by DPPH and FRAP assays in six plant-based milk alternatives. Different superscript letters indicate significant differences among plant-based milk alternatives within the same parameter (*p* < 0.05).

The total phenolic content (TPC), total flavonoid content (TFC), and antioxidant activities of six plant-based milk alternatives, including coconut, almond, cashew nut, soybean, peanut, and mung bean, are presented in Figure 2. These bioactive compounds are recognized for their health-promoting effects and are important quality indicators in functional food development. Among the samples, mung bean milk exhibited the highest TPC (309.09 µg GAE/mL) and TFC (304.63 µg QE/mL), significantly surpassing all others (Table S1). These results align with previous studies reporting that legumes, especially mung beans, are rich in phenolic acids and flavonoids [30]. Also, Peanut milk showed the second highest levels of TPC and TFC. Interestingly, some milks such as cashew nut and soybean, displayed higher TPC than TFC, whereas coconut, almond and peanut milks showed higher TFC than TPC. This variation has been reported in previous studies [19,31,32] and may be attributed to the differential sensitivity of the Folin–Ciocalteu method to specific flavonoid structures. Flavonoids, which possess different reducing capacities depending on their hydroxylation and glycosylation patterns [33], may react less effectively with the Folin–Ciocalteu reagent, potentially resulting in an underestimation of their actual concentration. Among the six samples, coconut milk exhibited the lowest levels of both TPC and TFC.

Antioxidant capacity measured by DPPH and FRAP assays showed similar trends. Mung bean milk demonstrated the highest radical scavenging activity (270.05 µg VCE/mL) and ferric reducing power (933.23 µg FeSO_4_/mL), indicating strong antioxidant potential (Figure 2 and Table S1). These results correspond with its high phenolic and flavonoid contents, which play a role in reducing oxidative stress [34,35]. Peanut and almond milks also exhibited substantial antioxidant activities, whereas coconut and soybean milks showed significantly lower values (Figure 2).

#### 3.1.3. Amino Acid Profiles of Plant-Based Milk Alternatives

**Figure 3:**
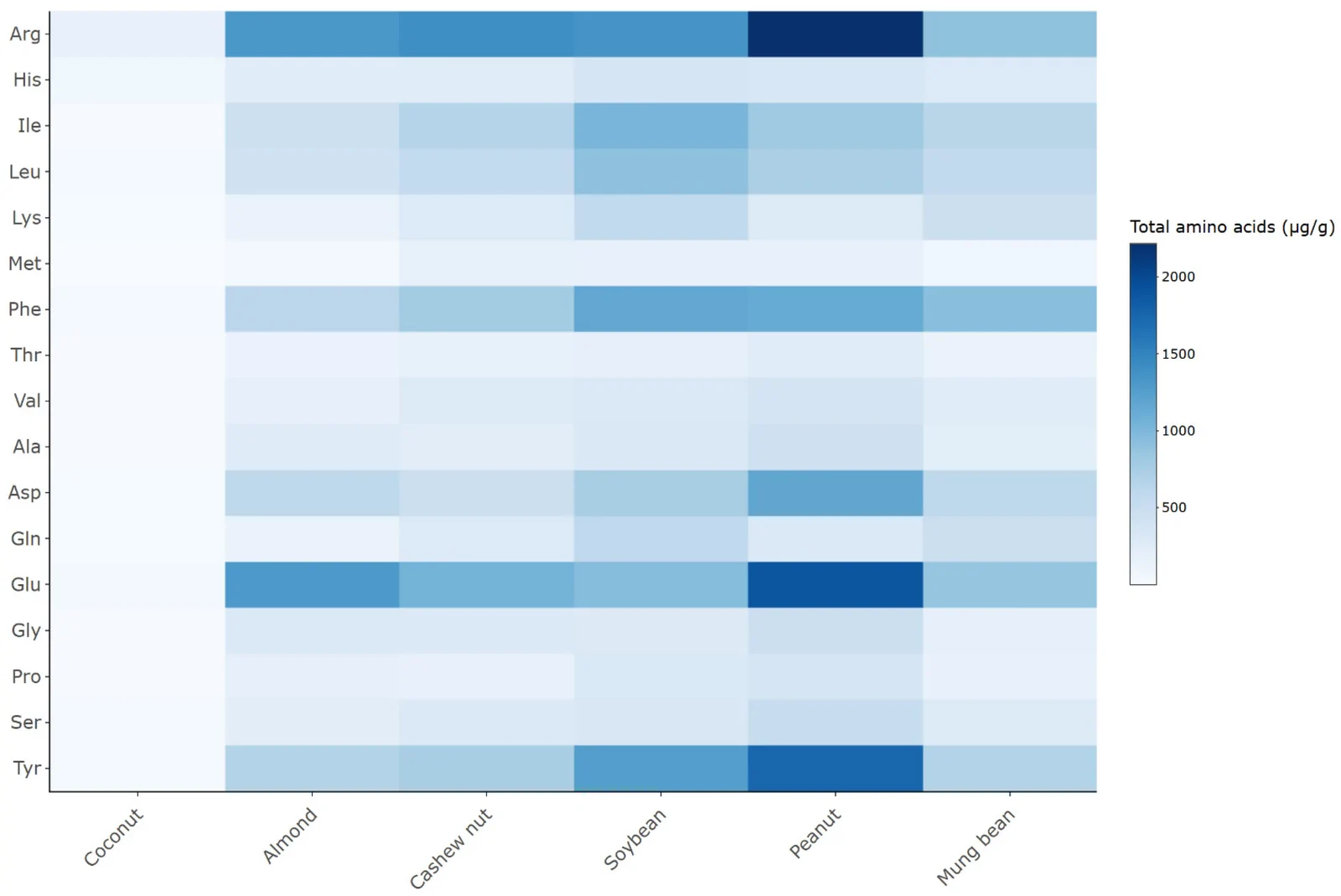
Heatmap of total amino acid content in six plant-based milk alternatives. Abbreviations: Arg: arginine, His: histidine, Ile: isoleucine, Leu: leucine, Lys: lysine, Met: methionine, Phe: phenylalanine, Thr: threonine, Val: valine, Ala: alanine, Asp: aspartic acid, Gln: glutamine, Glu: glutamic acid, Gly: glycine, Pro: proline, Ser: serine, and Tyr: tyrosine.

The amino acid composition of coconut, mung bean, soybean, cashew nut, almond, and peanut milk alternatives was visualized using a heatmap (Figure 3). The amino acid content data are presented in Table S2. Amino acids are key indicators of nutritional quality and protein functionality in food matrices [36]. Distinct variations in amino acid profiles were observed among the six plant-based milk alternatives. Overall, peanut milk contained the highest total amino acid content, followed by soybean milk. Regarding essential amino acids, peanut milk exhibited significantly higher levels of arginine, threonine and valine, with arginine being particularly prominent, whereas soybean milk showed significantly higher levels of histidine, isoleucine, leucine, lysine, and phenylalanine, compared to other samples (Figure 3 and Table S2). Arginine was especially abundant in the peanut, cashew nut, soybean, and almond milks. This finding is consistent with previous studies reporting high arginine levels in almond milk and peanut milks [37,38]. As a semi-essential amino acid, arginine serves as a key precursor of nitric oxide (NO), which supports vascular health by promoting vasodilation, enhancing blood flow, and providing cardioprotective effects [39].

For non-essential amino acids, peanut milk exhibited the highest concentrations across most amino acids, except for glutamine, which was most abundant in soybean milk. Glutamic acid, tyrosine, and aspartic acid were particularly abundant in peanut milk (Figure 3). In contrast, coconut milk contained the lowest levels of total, essential and non-essential amino acids, consistent with its well-documented macronutrient profile characterized by low protein and high lipid content [40,41]. Overall, variations in amino acid composition show how important it is to choose the right plant-based milk alternative based on the specific nutritional and functional needs in food product development.

### 3.2. Changes in Bioactive Compounds, Phenolic and Flavonoid Profiles, Antioxidant Activities, and Free Amino Acids of Plant-Based Milk Alternatives Following In Vitro Digestion

#### 3.2.1. Effect of Digestion on Bioactive Compounds and Antioxidant Activities in Six Plant-Based Milk Alternatives

Plant-based dairy alternatives have gained increasing interest due to the presence of bioactive constituents such as polyphenols and flavonoids, which are widely recognized for their antioxidant properties [15]. During digestion, bioactive compounds in plant-based milk alternatives may be influenced by the digestive process. In the present study, total phenolic content (TPC) significantly increased (*p* < 0.05) in all experimental samples (with enzymatic digestion) compared to their respective controls (no enzyme), especially in cashew nut, peanut, and almond milks (Table 1). The increase in TPC may result from the release of phenolic compounds that were entrapped within plant cell structures or bound to macromolecules in complex food matrices during digestion. Previous studies have demonstrated that enzymatic hydrolysis can effectively release phenolic compounds bound within the matrix, enhancing their solubility and availability in the supernatant [42,43].

In contrast to TPC, most plant-based milk alternatives showed no significant difference in total flavonoid content (TFC) following enzymatic digestion compared to the controls (no enzyme), except for mung bean milk, which exhibited a significant increase, and peanut milk, which showed a significant decrease in TFC after enzymatic digestion (Table 1). These variations observed in TFC among the plant-based milk alternatives may be explained by differences in their chemical compositions and interactions with other matrix components [18,44]. For example, protein–bioactive interactions are known to depend on protein structure, bioactive structure, matrix characteristics, and protein-to-bioactive ratio [45]. Such interactions may induce protein cross-linking and structural modifications [46], which could potentially affect enzymatic digestion and the release of certain bioactive compounds. In plant-based matrices, bioactive compounds may occur in free or bound forms, and enzymatic digestion may either release matrix-bound compounds, resulting in increased measurable flavonoid content, or reduce extractability, leading to lower flavonoid recovery. Moreover, certain flavonoids may exhibit different stability during enzymatic digestion [47]. Therefore, the observed increase in TFC may result from the release of flavonoids bound to other molecules, whereas the decrease may be attributed to reduced extractability and/or flavonoid degradation under digestive conditions.

Antioxidant activity, as measured by the DPPH radical scavenging assay, was significantly enhanced in all plant-based milk alternatives following enzymatic digestion compared to their respective control. The increase ranged from 2- to 4-fold for each sample relative to its control. These improvements are likely associated with the enhanced release of phenolic compounds [48]. FRAP assay results generally paralleled the DPPH findings, showing significant increases in most samples, except for coconut and mung bean milks. Despite their high DPPH activity, mung bean and coconut milks did not show a corresponding increase in FRAP. This divergence between the assays has been previously reported and may be attributed to differences in the electron-transfer mechanisms and redox potentials of the phenolic compounds involved [49].

#### 3.2.2. Profile of Individual Phenolic Acids and Flavonoids in Plant-Based Milk Alternatives After In Vitro Digestion

This study quantified individual phenolic acids and flavonoid compounds in control samples (no enzyme) and experimental samples (with enzymatic digestion) across six plant-based milks (Table 2). Total phenolic acid content increased significantly in all samples following enzymatic digestion (*p* < 0.05), consistent with the TPC in Table 1, which also showed elevated levels in digested samples compared to the controls. Several compounds that were absent or present in low amounts in the controls appeared or increased following enzymatic digestion, likely due to the action of digestive enzymes. In agreement with these findings, Djaoudene et al. (2021) reported that individual phenolics from certain fruit seeds were efficiently released following in vitro digestion [50].

For instance, gallic acid, which was absent in all controls, appeared in the experimental samples at concentrations ranging from 130.11 to 166.89 µg/mL. Protocatechuic acid, present only in trace amounts in a few controls (cashew nut and peanut milks), increased more than 200-fold after enzymatic digestion, ranging from 2370.49 to 2636.08 µg/mL. Syringic acid was detected only in digested samples of coconut, almond, cashew nut, and soybean milks. Interestingly gentisic acid was present in control peanut and mung bean milks and appeared in all experimental samples at higher levels, except in peanut milk, where it was lower than in the controls. The observed increases likely result from the release of bioactive bound to other molecules, whereas decreases may reflect phenolic degradation or reduced extractability under enzymatic digestive conditions. Since phenolic compounds can interact with other molecules and influence their structural and functional properties [45,46], these interactions may subsequently affect the release and extractability of phenolic compounds during digestion. Moreover, different phenolic compounds exhibit varying stability during in vitro digestion [51], which may contribute to differences in the measured concentrations of individual bioactive compounds. Ferulic, sinapic, and cinnamic acids were variably present and showed minimal or no significant increases, indicating that not all bound phenolics are equally susceptible to enzymatic release. Overall, total phenolic acid content increased significantly after enzymatic digestion, supporting the effectiveness of enzymatic hydrolysis in releasing matrix-bound polyphenols [52].

Interestingly, the summed concentrations of individual phenolic compounds in the experimental samples were higher than the TPC values determined by the Folin–Ciocalteu assay (Table 1 and Table 2). Since the Folin–Ciocalteu method provides only an estimate of total phenolic content rather than a direct quantification of phenolic compounds, this discrepancy may be related to the methodological characteristics of the assay. The Folin–Ciocalteu assay is based on an electron-transfer reaction and can be influenced by reaction conditions, such as pH, temperature, and reaction time, as well as food matrix effects and the reducing capacity of nonphenolic compounds [33]. In addition, TPC values were expressed as gallic acid equivalents, whereas protocatechuic acid and gentisic acid were the predominant individual phenolic compounds detected in the experimental samples (Table 2). Differences in the reactivity of phenolic compounds relative to gallic acid may therefore contribute to underestimation of TPC values, resulting in summed individual phenolic concentrations exceeding the measured TPC. Similar observations have also been reported in previous studies [53,54].

According to flavonoid profiles, overall total flavonoid content was also increased in cashew nut, soybean, peanut and mung bean milks after enzymatic digestion compared to the controls (no enzyme). However, the changes in flavonoid content were less pronounced than those observed for phenolic acids. When individual flavonoids were examined, myricetin showed nonsignificant difference across all samples. Quercetin and apigenin were consistently detected and generally exhibited only minor differences between control and experiment samples. These findings are consistent with the TFC results presented in Table 1, which showed that most samples exhibited no significant differences following enzymatic digestion, except for mung bean milk, which showed an increase, and peanut milk, which showed a decrease in the experimental samples compared with the controls. The discrepancy between the TFC results and the individual flavonoid profiles may be explained by the fact that only six flavonoids were quantified in this study. Other unmeasured flavonoid compounds may also be present, that have not been investigated. Rutin, a glycosylated quercetin derivative, was detected only in the experimental mung bean milk (113.24 µg/mL), whereas catechin was not detected in any of the samples. Additionally, kaempferol showed an increase in the experimental samples of cashew nut, soybean, peanut and mung bean milks compared with their respective controls. These results suggest that flavonoid release is influenced by both the composition of the plant matrix and the susceptibility to enzymatic cleavages during enzymatic digestion. Flavonoids can occur in various forms, including glycosides and aglycones [55], which may differ in their release, transformation, and stability under digestive conditions. Previous studies have shown that flavonoid glycosides may be broken down or undergo deglycosylation during digestion, leading to the formation of their corresponding aglycones, while O-glycosides, C-glycosides, and aglycone forms can differ in structural stability during in vitro digestion [47,56]. Therefore, the variable detection and recovery of individual flavonoids after digestion may be related to their chemical composition, molecular weight, subclass, esterification, glycosylation, and stability under digestive conditions [56,57].

Experimental samples (with enzymatic digestion) generally showed a significant enhancement in the extractability of phenolic acids and flavonoids in plant-based milks, particularly in cashew nut, soybean, peanut and mung bean milks. The detection of compounds that were previously undetected in certain control samples but appeared after enzymatic digestion, such as gallic acid, syringic acid, gentisic acid, protocatechuic acid, and rutin, highlights the potential of enzymatic digestion to enhance the release of bioactive compounds.

#### 3.2.3. Free Amino Acid Profiles in Plant-Based Milk Alternatives After In Vitro Digestion

**Figure 4:**
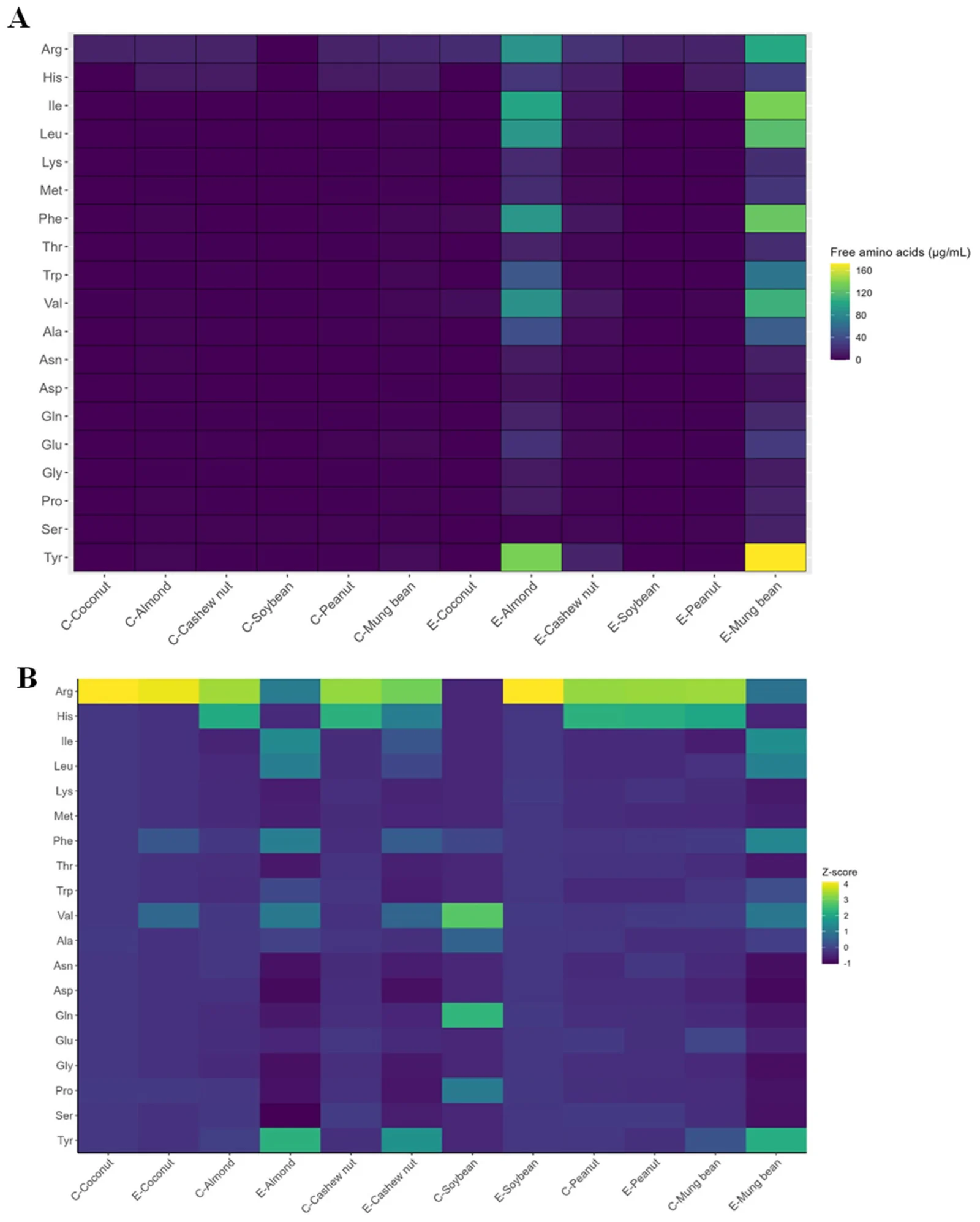
Heatmap analysis of (**A**) absolute concentrations of free amino acids and (**B**) z-score standardized free amino acid levels in control without enzyme treatment (indicated by the letter C) and experimental samples subjected to enzymatic digestion (indicated by the letter E) of six plant-based milks. Abbreviations: Arg: arginine, His: histidine, Ile: isoleucine, Leu: leucine, Lys: lysine, Met: methionine, Phe: phenylalanine, Thr: threonine, Trp: tryptophan, Val: valine, Ala: alanine, Asn: asparagine, Asp: aspartic acid, Gln: glutamine, Glu: glutamic acid, Gly: glycine, Pro: proline, Ser: serine, and Tyr: tyrosine.

This study quantified free amino acids in control samples (no enzyme) and experimental samples (with enzymatic digestion) across six plant-based milks (Figure 4A,B). The free amino acid content data are presented in Table S3. Overall, the experimental samples subjected to enzymatic digestion showed an increase in free amino acid levels compared with the control samples, particularly in almond, cashew nut, and mung bean milks (Figure 4A and Table S3), indicating that these plant-based milks were more responsive to in vitro enzymatic digestion, resulting in enhanced release of amino acids. Meanwhile coconut, soybean, and peanut milks showed relatively minor differences between the control and experimental samples. Interestingly, in soybean milk arginine showed a marked increase in the experimental samples, suggesting that enzymatic treatment may selectively promote the release of certain amino acids while others remain relatively unchanged (Figure 4A and Table S3). A z-score heatmap (Figure 4B) further illustrated the relative distribution patterns of free amino acids by showing how individual amino acid levels deviated from their mean values within the sample. The heatmap supported the quantitative data (Figure 4A), demonstrating distinct amino acid patterns between experimental and control samples, particularly in almond, cashew nut, and mung bean milks. In contrast, coconut and peanut milks exhibited similar patterns between treatments, indicating minimal changes in their amino acid profiles, whereas soybean milk showed a slightly different pattern due to the relatively high abundance of arginine in the experimental sample compared with the control.

The differences in free amino acid responses among plant-based milk alternatives following enzymatic digestion may be attributed to variations in protein structure and interactions with other matrix components, which influence enzyme accessibility and protein digestibility. Protein hydrolysis during digestion releases peptides and free amino acids, resulting in increased amino acid concentrations after enzymatic treatment [58]. However, the extent of amino acid release can vary depending on the protein source, the structure and amino acid composition, processing conditions, and interactions with matrix components [45,59,60]. For instance, phytate and dietary fibers can reduce protein digestibility and bioavailability by forming stable complexes with metal ions and proteins [60], while protein–phenolic interactions may alter protein structure and hinder enzymatic access [45]. These factors may explain the distinct digestion responses observed across the different plant-based milks in this study.

## 4. Conclusions

This study demonstrated that plant-based milk alternatives differ substantially in their bioactive compound composition, antioxidant capacity, and amino acid profiles, and that these properties are significantly influenced by in vitro digestion. Generally, enzymatic digestion enhanced the release of phenolic compounds and increased antioxidant activity across all plant-based milks, supporting the role of digestive processes in improving the extractability of matrix-bound bioactive compounds. However, total flavonoid responses varied among samples, indicating that flavonoid stability and extractability depend on matrix composition and molecular characteristics. Similarly, free amino acid release differed among plant sources, with almond, cashew nut, and mung bean milks showing greater responsiveness to digestion compared with coconut, soybean, and peanut milks. These differences are likely associated with variations in protein structure and interactions with matrix components that influence protein digestibility. Further studies are needed to investigate the mechanisms underlying the effects of composition and food matrix characteristics on digestibility and the bioactivity of bioactive compounds.

Overall, the findings highlight the importance of considering both food matrix composition and digestive conditions when evaluating the nutritional and functional potential of plant-based milk alternatives. This study provides new insights into the behavior of individual bioactive compounds and amino acids during digestion and supports the development of plant-based beverages with improved functional and nutritional properties. However, this study was limited to a static in vitro digestion model and did not include sensory evaluation, consumer acceptance testing, or verification using multiple raw material varieties or sources. Further studies using dynamic digestion models, multiple batches or varieties of raw materials, and evaluations of sensory and product-quality attributes are needed to confirm the applicability of these findings under broader formulation and product-development conditions.

## Figures and Tables

**Table 1:** The total phenolic content (TPC), total flavonoid content (TFC), and antioxidant activities assessed by DPPH and FRAP assays in six plant-based milk alternatives following control sample (no enzyme) and experimental sample (with enzyme) treatment.

Treatment	Plant-Based Milks	TPC (mg GAE/mL)	TFC (mg QE/mL)	DPPH (µg Vitamin C/mL)	FRAP (mg FeSo4/mL)
Control samples (no enzyme)	Coconut	0.15 ± 0.12 ^d^	13.31 ± 0.80 ^b^	129.93 ± 7.80 ^i^	9.50 ± 0.04 ^d^
	Almond	0.18 ± 0.05 ^d^	13.52 ± 0.10 ^b^	215.53 ± 13.51 ^h^	9.54 ± 0.03 ^d^
	Cashew nut	0.32 ± 0.06 ^d^	9.30 ± 0.46 ^b^	202.01 ± 13.51 ^h^	9.56 ± 0.02 ^d^
	Soybean	0.34 ± 0.05 ^d^	13.23 ± 0.92 ^b^	278.60 ± 20.64 ^g^	9.63 ± 0.17 ^d^
	Peanut	0.31 ± 0.03 ^d^	15.33 ± 0.97 ^a^	332.66 ± 20.64 ^f^	9.59 ± 0.02 ^d^
	Mung bean	0.32 ± 0.06 ^d^	13.42 ± 0.36 ^b^	427.26 ± 20.64 ^e^	9.58 ± 0.14 ^d^
Experimental samples (with enzyme)	Coconut	2.37 ± 0.10 ^c^	13.79 ± 0.54 ^b^	431.77 ± 27.03 ^e^	9.71 ± 0.04 ^d^
	Almond	3.58 ± 0.13 ^ab^	13.99 ± 0.11 ^b^	485.83 ± 27.03 ^d^	11.21 ± 0.13 ^ab^
	Cashew nut	3.91 ± 0.39 ^a^	13.79 ± 0.59 ^b^	738.11 ± 41.29 ^b^	10.98 ± 0.33 ^b^
	Soybean	3.52 ± 0.37 ^b^	13.45 ± 0.35 ^b^	566.92 ± 27.03 ^c^	10.04 ± 0.08 ^c^
	Peanut	3.66 ± 0.22 ^ab^	13.63 ± 1.35 ^b^	711.08 ± 15.61 ^b^	11.24 ± 0.19 ^a^
	Mung bean	3.29 ± 0.35 ^b^	16.00 ± 0.31 ^a^	954.34 ± 41.29 ^a^	9.52 ± 0.20 ^d^

Data are expressed as mean ± SD of triplicate measurements. Different superscript letters within the same column indicate significant differences among all control samples (no enzyme) and experimental samples (with enzymatic digestion) for each parameter (*p* < 0.05).

**Table 2:** Phenolic acid and flavonoid HPLC of six plant-based milk alternatives following control sample (no enzyme) and experimental sample (with enzyme) treatment.

Parameter	Control Samples (No Enzyme)						Experimental Samples (With Enzyme)					
	Coconut	Almond	Cashew Nut	Soybean	Peanut	Mung Bean	Coconut	Almond	Cashew Nut	Soybean	Peanut	Mung Bean
Phenolic acid content (µg/mL)												
GA ^ns^	ND	ND	ND	ND	ND	ND	158.40 ± 51.89	152.52 ± 50.52	166.89 ± 40.85	145.93 ± 30.55	135.66 ± 36.68	130.11 ± 10.18
PCCA	ND	ND	10.41 ± 0.67 ^d^	ND	7.26 ± 0.76 ^d^	ND	2572.77 ± 153.34 ^ab^	2382.65 ± 109.34 ^c^	2575.50 ± 126.30 ^ab^	2370.49 ± 80.15 ^c^	2636.08 ± 130.77 ^a^	2475.51 ± 28.48 ^bc^
p-HBA	ND	ND	ND	ND	ND	ND	ND	ND	ND	ND	ND	ND
VA	ND	ND	ND	ND	ND	ND	ND	ND	ND	ND	ND	ND
CA	ND	ND	ND	ND	ND	2.30 ± 0.21	ND	ND	ND	ND	ND	ND
SyA	ND	ND	ND	ND	ND	ND	7.43 ± 1.84 ^ab^	6.53 ± 0.75 ^bc^	5.97 ± 0.46 ^c^	8.48 ± 0.91 ^a^	ND	ND
p-CA	ND	ND	ND	ND	ND	38.19 ± 0.77	ND	ND	ND	0.96 ± 0.09 ^b^	ND	38.19 ± 0.77 ^a^
FA	3.75 ± 1.11 ^a^	1.78 ± 1.16 ^b^	2.60 ± 2.03 ^ab^	3.53 ± 0.93 ^ab^	3.53 ± 0.28 ^ab^	3.81 ± 1.50 ^a^	2.65 ± 0.09 ^ab^	3.31 ± 0.70 ^ab^	3.42 ± 0.83 ^ab^	3.39 ± 0.63 ^ab^	3.08 ± 0.03 ^ab^	2.42 ± 0.51 ^ab^
SA	17.72 ± 0.19 ^de^	38.52 ± 11.79 ^bcd^	18.95 ± 8.08 ^de^	17.73 ± 3.77 ^de^	41.00 ± 7.83 ^bc^	46.58 ± 16.14 ^b^	11.87 ± 0.35 ^e^	49.14 ± 17.10 ^b^	23.48 ± 4.62 ^cde^	54.55 ± 19.45 ^ab^	72.82 ± 17.78 ^a^	12.55 ± 0.25 ^e^
CAN	39.59 ± 0.31b ^c^	42.22 ± 12.96 ^ab^	23.85 ± 20.86 ^bc^	21.31 ± 5.76 ^c^	23.41 ± 3.80 ^c^	58.35 ± 19.01 ^a^	27.87 ± 0.28 ^bc^	22.48 ± 5.91 ^c^	21.52 ± 6.80 ^c^	37.29 ± 0.61 ^bc^	30.9 ± 2.02 ^bc^	30.44 ± 5.80 ^bc^
GTA	ND	ND	ND	ND	3543.15 ± 623.63 ^a^	462.24 ± 145.66 ^e^	3766.61 ± 379.42 ^a^	2944.85 ± 233.28 ^cd^	3391.95 ± 292.65 ^abc^	2977.73 ± 275.13 ^bcd^	2448.93 ± 562.84 ^d^	3414.53 ± 65.96 ^abc^
Total	61.06 ± 0.41 ^f^	82.52 ± 5.03 ^f^	55.81 ± 7.98 ^f^	42.57 ± 1.98 ^f^	3618.35 ± 248.20 ^d^	645.72 ± 42.18 ^e^	6547.60 ± 131.43 ^a^	5561.48 ± 79.59 ^c^	6188.73 ± 101.02 ^b^	5598.82 ± 88.51 ^c^	5327.47 ± 200.74 ^c^	6196.33 ± 22.39 ^b^
Flavonoid content (µg/mL)												
RT	ND	ND	ND	ND	ND	ND	ND	ND	ND	ND	ND	113.24 ± 1.26
CTC	ND	ND	ND	ND	ND	ND	ND	ND	ND	ND	ND	ND
QCT	76.87 ± 12.09 ^a^	74.04 ± 10.24 ^ab^	74.07 ± 8.12 ^ab^	80.44 ± 4.17 ^a^	54.73 ± 15.31 ^c^	60.33 ± 3.64 ^bc^	79.52 ± 10.76 ^a^	78.78 ± 12.79 ^a^	85.90 ± 0.57 ^a^	80.67 ± 0.68 ^a^	78.84 ± 1.68 ^a^	71.75 ± 1.79 ^ab^
APG	134.68 ± 4.64 ^ab^	62.37 ± 10.49 ^b^	114.09 ± 91.86 ^ab^	140.57 ± 55.41 ^a^	107.00 ± 52.87 ^ab^	125.09 ± 20.24 ^ab^	127.21 ± 7.81 ^ab^	116.59 ± 14.89 ^ab^	144.99 ± 26.99 ^a^	104.45 ± 3.33 ^ab^	104.02 ± 4.77 ^ab^	127.73 ± 0.20 ^ab^
MCT ^ns^	75.34 ± 1.82	186.00 ± 92.66	65.31 ± 4.89	73.59 ± 2.11	63.31 ± 3.59	66.74 ± 5.02	81.19 ± 0.82	84.61 ± 4.61	81.81 ± 1.27	86.98 ± 4.48	87.36 ± 2.08	85.55 ± 2.65
KFR	136.96 ± 1.93 ^bc^	97.65 ± 61.99 ^bcd^	62.47 ± 23.42 ^d^	132.62 ± 56.75 ^bcd^	117.11 ± 49.19 ^bcd^	64.11 ± 5.04 ^d^	138.08 ± 2.27 ^bc^	133 ± 2.31 ^bcd^	165.15 ± 54.33 ^b^	338.93 ± 46.73 ^a^	317.18 ± 5.64 ^a^	136.93 ± 1.78 ^bc^
Total	423.85 ± 4.83 ^c^	420.06 ± 40.64 ^bc^	315.94 ± 40.67 ^d^	427.22 ± 30.58 ^bc^	342.15 ± 24.52 ^d^	255.94 ± 7.85 ^e^	426.00 ± 4.67 ^c^	412.98 ± 6.12 ^c^	477.85 ± 25.52 ^b^	611.03 ± 22.01 ^a^	587.40 ± 1.96 ^a^	449.65 ± 1.02 ^c^

Data are expressed as mean ± standard deviation (SD); ND, not detected; ns, not significant. Different superscript letters within the same row indicate significant differences among all control samples (no enzyme) and experimental samples (with enzymatic digestion) for each compound (*p* < 0.05); gallic acid (GA), protocatechuic acid (PCCA), p-hydroxybenzoic acid (p-HBA), vanillic acid (VA), caffeic acid (CA), syringic acid (SyA), p-coumaric acid (p-CA), ferulic acid (FA), sinapic acid (SA), cinnamic acid (CAN), gentisic acid (GTA), rutin (RT), catechin (CTC), quercetin (QCT), apigenin (APG), myricetin (MCT), kaempferol (KFR).

## Data Availability

The original contributions presented in this study are included in the article/Supplementary Materials. Further inquiries can be directed to the corresponding author.

## Supplementary Materials

The following supporting information can be downloaded at: https://www.mdpi.com/article/10.3390/foods15162907/s1, Table S1: Total phenolic content (TPC), total flavonoid content (TFC), and antioxidant activities measuring by DPPH and FRAP in six plant-based milk alternatives; Table S2: Content of total amino acids in the six plant-based milk alternatives; Table S3: Free amino acid of six plant-based milk alternatives following control samples (no-enzyme) and experimental samples (with enzyme) treatment.
