# Supplementary material for: In Vitro Digestion Influences Bioactive Compound Profiles, Antioxidant Activity, and Free Amino Acid Release in Plant-Based Milk Alternatives
Source: Foods. 2026 Aug 19;15(16):2907. doi: 10.3390/foods15162907 (PMC13511869; doi:10.3390/foods15162907)
Supplement: Supplementary file 1 [file foods-15-02907-s001.zip › foods-4488746-supplementary.pdf]

In Vitro Digestion Influences Bioactive Compound Profiles, Antioxidant Activity, and Free Amino Acid Release in Plant-Based Milk Alternatives

Nonthiwat Taesuk, Theeraphan Chumroenphat, Nidthaya Seephua, Manmanut Srikaew, Sirithon Siriamornpun, Daniela Barile, Apichaya Bunyatratchata

Supplementary Tables

Table S1. Total phenolic content (TPC), total flavonoid content (TFC), and antioxidant activities measuring by DPPH and FRAP in six plant-based milk alternatives.

Parameter	Plant-based milks					
	Coconut	Almond	Cashew nut	Soybean	Peanut	Mung bean
TPC (µg GAE/mL)	25.54±1.02^{e}	145.30±6.88^{d}	141.56±4.89^{d}	158.19±0.83^{c}	203.16±6.38^{b}	309.09±12.92^{a}
TFC (µg QE/mL)	47.59±3.57^{e}	264.63±6.41^{c}	120.56±4.45^{d}	115.00±3.33^{d}	287.96±4.49^{b}	304.63±11.56^{a}
DPPH (µg VCE/mL)	63.17±0.72^{d}	98.87±1.82^{c}	103.21±1.67^{c}	65.34±1.25^{d}	149.76±1.10^{b}	270.05±5.79^{a}
FRAP (µg $FeSO_4$/mL)	117.90±1.81^{e}	387.69±3.25^{b}	265.19±1.66^{c}	196.44±3.90^{d}	384.77±1.91^{b}	933.23±9.55^{a}

Data are expressed as mean ± SD. Mean values in the same row with different superscript letters are significantly different ($p < 0.05$).

Table S2. Content of total amino acids in the six plant-based milk alternatives.

Parameter	Plant-based milks (µg/g)					
	Coconut	Almond	Cashew nut	Soybean	Peanut	Mung bean
Essential AA						
Arginine	165.57±0.66^{e}	1325.22±5.36^{c}	1411.37±29.16^{b}	1357.88±8.46^{c}	2212.85±49.33^{a}	911.52±11.96^{d}
Histidine	80.51±0.12^{f}	244.92±0.58^{e}	256.12±2.96^{d}	368.12±4.82^{a}	342.97±4.74^{b}	298.48±3.85^{c}
Isoleucine	10.86±0.65^{f}	466.90±4.19^{e}	665.24±11.11^{c}	1027.25±5.29^{a}	824.69±4.57^{b}	647.38±2.94^{d}
Leucine	20.90±1.08^{e}	420.45±2.18^{d}	561.42±5.46^{c}	914.66±5.56 ^{a}	735.91±3.06^{b}	567.61±2.73^{c}
Lysine	18.65±0.37^{f}	139.81±1.28^{e}	296.49±5.51^{c}	571.01±2.39^{a}	288.31±1.36^{d}	472.27±4.61^{b}
Methionine	1.20±0.16^{f}	37.02±1.40^{e}	178.71±3.31^{a}	161.67±1.61^{c}	165.98±2.58^{b}	92.13±0.87^{d}
Phenylalanine	33.48±2.55^{f}	626.69±3.74^{e}	791.43±6.63^{d}	1168.29±3.01^{a}	1131.65±6.77^{b}	939.59±6.77^{c}
Threonine	6.62±0.56^{e}	130.83±10.83^{d}	184.11±1.06^{c}	210.59±18.09^{b}	250.24±2.91^{a}	140.86±2.01^{d}
Tryptophan	ND	ND	ND	ND	ND	ND
Valine	10.84±0.84^{e}	200.39±2.85^{d}	291.28±2.39^{b}	305.86±14.11^{b}	384.14±5.84^{a}	249.14±5.03^{c}
Total essential AA	384.63±1.40^{f}	3592.23±3.45^{e}	4636.17±6.89^{c}	6085.33±7.11^{b}	6336.74±3.02^{a}	4318.98±14.10^{d}
Non-Essential AA						
Alanine	10.92±0.29^{e}	262.38±10.02^{c}	234.13±2.07^{d}	318.09±9.55^{b}	462.32±9.25^{a}	220.94±11.62^{d}
Asparagine	ND	ND	ND	ND	ND	ND
Aspartic acid	14.34±0.10^{e}	613.12±10.55^{c}	491.08±10.56^{d}	757.40±0.96^{b}	1177.23±43.10^{a}	614.51±9.73^{c}
Cysteine	ND	ND	ND	ND	ND	ND
Glutamine	19.66±0.24^{e}	144.75±0.79^{d}	295.74±4.85^{c}	592.84±5.37^{a}	301.01±1.36^{c}	487.24±1.15^{b}
Glutamic acid	40.40±4.36^{f}	1303.28±26.60^{b}	1054.65±13.46^{c}	965.64±85.22^{d}	1889.31±78.29^{a}	870.68±3.52^{e}
Glycine	9.00±0.91^{e}	303.23±7.71^{b}	302.37±9.69^{b}	279.11±6.45^{c}	472.12±12.09^{a}	195.71±8.16^{d}
Proline	6.54±0.25^{e}	197.92±6.86^{c}	166.62±6.67^{d}	323.50±3.86^{b}	377.75±7.42^{a}	165.51±3.17^{d}
Serine	22.10±0.49^{f}	232.01±0.30^{e}	301.80±6.70^{c}	336.06±3.96^{b}	535.70±6.52^{a}	286.02±8.03^{d}
Tyrosine	28.24±1.46^{e}	685.24±8.04^{d}	759.17±26.48^{c}	1276.25±21.29^{b}	1747.40±3.22^{a}	683.51±4.69^{d}
Total non-essential AA	151.20±1.00^{f}	3,741.93±7.95^{c}	3,605.56±10.06^{d}	4,848.89±17.08^{b}	6,962.84±20.16^{a}	3,524.12±6.26^{e}
Total AA	499.83±1.21^{f}	7,334.16±5.70^{e}	8,241.73±8.48^{c}	10,934.22± 12.10^{b}	13,299.58±11.59^{a}	7,843.10±10.18^{d}

Data are expressed as mean ± SD; ND: Not detected. Mean values in the same row with different superscript letters are significantly different ($p < 0.05$).

Table S3. Free amino acid of six plant-based milk alternatives following control samples (no- enzyme) and experimental samples (with enzyme) treatment.

	**Control samples (no- enzyme) µg/mL**						**Experimental samples (with enzyme) µg/mL**					
	**Coconut**	**Almond**	**Cashew nut**	**Soybean**	**Peanut**	**Mung bean**	**Coconut**	**Almond**	**Cashew nut**	**Soybean**	**Peanut**	**Mung bean**
Arg	14.37±0.00	15.58±0.18	14.40±0.02	ND	14.39±0.03	16.37±0.60	19.80±5.43	88.48±17.17	23.54±5.35	14.38±0.01	15.07±0.24	104.01±4.27
His	ND	10.37±0.01	10.36±0.01	ND	10.38±0.04	10.84±0.30	ND	25.57±3.29	12.33±1.40	ND	10.50±0.12	30.11±0.25
Ile	ND	ND	ND	ND	ND	ND	ND	100.24±30.58	7.53±5.27	ND	ND	136.25±6.41
Leu	ND	0.31±0.12	ND	ND	ND	1.35±0.89	ND	89.72±26.21	5.95±5.25	ND	ND	120.98±6.04
Lys	ND	0.43±0.10	0.17±0.00	ND	0.22±0.04	0.94±0.29	ND	17.74±3.08	2.44±1.73	ND	0.48±0.19	20.07±0.72
Met	ND	0.39±0.06	ND	ND	0.21±0.04	0.77±0.33	ND	18.96±3.70	2.58±1.81	ND	ND	24.87±1.44
Phe	ND	1.26±0.14	0.06±0.02	0.04±0.01	0.60±0.29	1.93±0.12	ND	89.59±26.11	8.21±5.76	0.04±0.00	0.72±0.37	127.66±4.57
Thr	ND	0.68±0.08	0.44±0.00	ND	0.54±0.08	1.00±0.21	ND	14.66±2.18	2.20±1.31	ND	0.53±0.08	19.24±0.75
Trp	ND	0.52±0.04	0.56±0.07	ND	ND	1.60±0.11	ND	46.65±17.75	1.80±2.21	ND	ND	65.98±3.59
Val	ND	1.26±0.05	0.30±0.00	0.23±0.01	0.66±0.25	2.04±0.46	ND	85.30±16.93	9.34±6.89	0.26±0.02	0.95±0.39	109.72±2.36
Ala	ND	1.20±0.03	0.57±0.06	0.07±0.01	0.72±0.35	1.08±0.00	ND	41.44±5.92	3.62±2.90	0.08±0.01	0.21±0.09	51.15±1.44
Asn	ND	1.21±0.12	ND	ND	ND	0.89±0.15	ND	10.01±1.34	1.79±0.92	ND	0.80±0.04	12.07±0.25
Asp	ND	0.80±0.12	0.12±0.01	ND	0.13±0.02	0.41±0.01	ND	5.90±1.09	0.70±0.56	ND	0.10±0.02	6.99±0.08
Gln	ND	0.49±0.09	0.21±0.01	0.20±0.00	0.31±0.08	0.85±0.23	ND	14.06±1.46	2.53±1.43	0.20±0.00	0.32±0.05	17.55±0.59
Glu	ND	0.61±0.05	0.62±0.07	ND	0.94±0.29	2.76±0.21	ND	22.53±5.63	3.08±2.17	ND	0.34±0.03	27.91±2.14
Gly	ND	0.38±0.06	0.22±0.02	ND	0.34±0.17	0.79±0.12	ND	9.71±0.54	1.37±1.02	ND	0.23±0.04	11.19±0.72
Pro	ND	1.17±0.24	0.32±0.07	0.11±0.01	0.37±0.14	0.96±0.23	ND	10.74±2.82	1.12±0.78	0.11±0.00	0.22±0.05	14.41±0.59
Ser	ND	1.22±0.02	1.03±0.02	ND	0.97±0.01	1.08±0.01	ND	1.02±0.07	1.92±0.77	ND	0.98±0.02	13.20±0.10
Tyr	ND	1.73±0.29	0.26±0.02	ND	0.79±0.30	3.74±1.14	ND	136.24±33.41	14.88±13.06	ND	0.33±0.10	172.31±9.91
TAA	14.91±5.69	39.61±3.93	29.64±4.15	1.77±0.28	31.57±4.11	49.40±4.03	27.97±7.52	828.56±39.83	106.93±5.77	15.24±4.98	31.78±4.27	1,085.67±51.84

Data are expressed as mean ± SD; ND: Not detected. Abbreviations: Arg: Arginine, His: Histidine, Ile: Isoleucine, Leu: Leucine, Lys: Lysine, Met: Methionine, Phe: Phenylalanine, Thr: Threonine, Trp: Tryptophan, Val: Valine, Ala: Alanine, Asn: Asparagine, Asp: Aspartic acid, Gln: Glutamine, Glu: Glutamic acid, Gly: Glycine, Pro: Proline, Ser: Serine, and Tyr: Tyrosine
